# Wallerian Degeneration in Central Nervous System: Dynamic Associations between Diffusion Indices and Their Underlying Pathology

**DOI:** 10.1371/journal.pone.0041441

**Published:** 2012-07-19

**Authors:** Wen Qin, Min Zhang, Yueshan Piao, Deyu Guo, Zixin Zhu, Xin Tian, Kuncheng Li, Chunshui Yu

**Affiliations:** 1 Department of Radiology, Tianjin Medical University General Hospital, Tianjin, China; 2 Department of Radiology, Xuanwu Hospital, Capital Medical University, Beijing, China; 3 Department of Pathology, Xuanwu Hospital, Capital Medical University, Beijing, China; 4 Department of Experimental Animal, Xuanwu Hospital, Capital Medical University, Beijing, China; Stanford University School of Medicine, United States of America

## Abstract

**Background:**

Although diffusion tensor imaging has been used to monitor Wallerian degeneration, the exact relationship between the evolution of diffusion indices and its underlying pathology, especially in central nervous system, remains largely unknown. Here we aimed to address this question using a cat Wallerian degeneration model of corticospinal tract.

**Methodology/Principal Findings:**

Twenty-five domestic mature Felis catus were included in the present study. The evolution of diffusion indices, including mean diffusivity (MD), fractional anisotropy (FA), primary (λ1) and transverse eigenvalues (λ23) of the degenerated corticospinal tract, were observed at baseline (before modeling) and at 2, 4, 6, 8, 10, 15, 20, 25, 30, 45 and 60 days after modeling in 4 cats. Pathological examinations were performed at eight time points mentioned above. Wallerian degeneration can be detected as early as the 2nd day after modeling by both diffusion tensor imaging and pathology. According to the evolution of diffusion indices, Wallerian degeneration can be classified into 2 stages. During the early stage (within 8 days after modeling), progressive disintegration of axons and myelin sheaths underlies the decreases in FA and λ1 and the increase in λ23. However, during the late stage (after 8 days), the gradual increases in FA, MD and λ1 and the unchanged λ23 seem to be a comprehensive reflection of the pathological processes including microglia activation, myelin clearance, and astrocytosis.

**Conclusions/Significance:**

Our findings help the understanding of the altered diffusion indices in the context of pathology and suggest that diffusion tensor imaging has the potential to monitor the processes of Wallerian degeneration in the central nervous system *in vivo* after acute damage.

## Introduction

The stereotyped process of degenerative events that occurs in distal axon after injury of the proximal parts of a neuron is known as Wallerian degeneration (WD) [1]. This pathological process begins with a rapid axonal disintegration and breakdown of myelin sheath, then activation of microglia, with subsequent clearance of tissue debris and gliosis [2], [3]. WD occurs in many diseases of the central nervous system (CNS), such as trauma [4], [5], stroke [6], [7], [8], multiple sclerosis [9], [10], [11] and Alzheimer's disease [12], [13], [14], etc. Numerous pieces of evidence suggest that WD is one of the major causes for the nonreversible functional deficiency in these diseases [5], [6], [15], [16], [17]. Consequently, using a non-invasive approach to early detect and characterize WD in CNS is clinically important, which may help to monitor the progress of axonal degeneration [15], [18], to early predict the prognosis of functional deficiency [6], [7], [19] and to assess the outcomes of therapeutic strategies on axonal degeneration [20], [21], [22].

Diffusion tensor imaging (DTI) measures the random motion of water molecules and has the potential to *in vivo* detect changes in microscopic architectures in brain white matter (WM) [23]. The DTI-derived indices include mean diffusivity (MD) and fractional anisotropy (FA), which are commonly used to quantify the average amplitude and the directionality of molecular motion, respectively [17], [24], [25]. Besides, the primary (λ1) and transverse eigenvalues (λ23) are also popularly used to reflect the diffusivities along the maximal and perpendicular directions, respectively [26], [27].

The dynamic evolution of diffusion indices in degenerated WM fiber tracts has been delineated previously. In a one-year follow-up study of stroke patients, Yu et al reported that: (1) WD can be detected at the second week with sharply decreased FA and λ1, and increased λ23; (2) from 2 week to 3 month, MD slightly increased accompanied by decreased FA, increased λ23 and unchanged λ1; and (3) all diffusion indices maintained a relatively stable level after 3 months [6]. Their findings were consistent with previous studies, using either a single or a multiple time-point data [15], [18], [19], [28]. However, these studies cannot answer the exact relationship between the evolution of diffusion indices and its underlying pathologic processes in WD since pathological data cannot be obtained from clinical observations of living patients.

In order to answer the question, a research group had studied the degenerated visual pathway of mice, and found that the detectable decrease of λ1 (3 days after injury) was earlier than the rise of λ23 (5 or 9 days), which were consistent with the time course of decreased phosphorylated neurofilament and myelin basic protein, respectively. Then they proposed that the λ1 represented axonal degeneration whereas the λ23 denoted the myelin clearance [29]. However, a recent study had reported an inconsistent results that both the reduced λ1 and the increased λ23 could be found in degenerated fibers at day 3 after unilateral dorsal root axotomy of the spinal cord [30]. The authors ascribed the alterations of λ1 and λ23 at the initial stage of WD to axon degeneration. Thus a further study is needed to elucidate the exact relationship between the evolution of diffusion indices and its underlying pathologic processes in WD.

In the present study, we made substantial improvements based on previous studies. Firstly, the observation time (60 days) is much longer and the time points (12 for DTI and 8 for pathology) are much denser than previous studies [27], [30], [31]. Secondly, much more pathological processes were observed in the present study, such as the axonal degeneration, microglia activation, debris clearance, and astrocytosis. Finally, we focused on the segment of the degenerated CST within the brain, one of the most important brain WM fiber tracts, which is commonly attacked by a variety of brain disorders. With these improvements, we aimed to elucidate the relationship between the evolution of diffusion indices and its underlying pathological processes in degenerated CST.

## Materials and Methods

### Animal modeling

**Figure 1:**
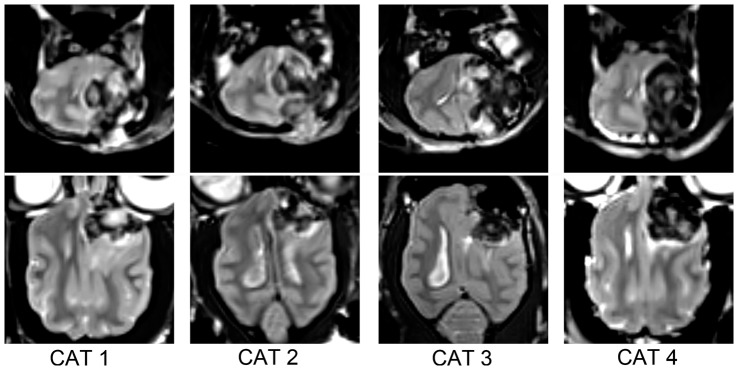
T2 weighted images of cat's brain at 8^th^ day after modeling. The cortical origins of left CST including left frontal region, precentral region and postcentral region were removed to establish the Wallerian degeneration model.

Twenty-five domestic mature cats (Felis catus; 12 males and 13 females; 1–2 years old; 3–5 kg) were included in the present study. Experimental procedures were approved by the Animal Care and Use Committee of Capital Medical University of China. All cats were free from motor function deficiency and did not show any brain lesions as revealed by conventional MRI. After baseline DTI scan, the animals were anesthetized by intramuscular injection of atropine (0.05 mg/kg), ketamine (25 mg/kg) and diazepam (0.5 mg/kg). Then the left frontoparietal cortex was exposed, and the cortical origins of left CST including left frontal region, precentral region and postcentral region [32] (**Fig. 1**) were excised to establish the WD model.

Four modeled cats were selected to study the dynamic evolution of diffusion indices in the degenerated CST at 12 sequential time points (TPs), including 0 (before modeling), 2, 4, 6, 8, 10, 15, 20, 25, 30, 45 and 60 days after modeling. After DTI scan at the last TP, they were prepared for pathological examination. The remaining 21 cats were randomly divided into 7 subgroups with 3 cats per group to investigate the pathological changes of the degenerated CST at 2, 4, 6, 10, 20, 30, and 45 days after modeling, respectively.

### DTI examination

A 3.0 Tesla MRI scanner (Trio Tim system; Siemens Magnetom, Erlangen, Germany) was used to acquire the DTI data. The scanner was equipped with a high-performance gradient system with 45 mT/m of maximum gradient strength and 200 T/m/s of maximum slew rate. An eight-channel transmitting-receiving knee coil was used to generate and receive MR signals since its inner diameter was comparable with the cat's head. After anesthesia, animals were fixed on a custom-made MRI-compatible device at the supine position.

A set of three dimensional T2 weighted (T2W) images was firstly acquired to localize the DTI scan [33]. Then the DTI data were obtained using a twice-refocused spin echo single-shot echo-planar diffusion tensor imaging (SS-EP-DTI) sequence by which eddy current artifacts were substantially eliminated [34]. The diffusion gradient encoding scheme included 30 non-collinear diffusion gradients with a b value of 1000 s/mm^2^ and one non-diffusion-weighted image (b = 0 s/mm^2^). Integrated parallel acquisition technique (iPAT) with generalized auto-calibrating partially parallel acquisition (GRAPPA) algorithm was also used to reduce image distortion from susceptibility (acceleration factor = 4, reference lines = 24). The other parameters were: repetition time/echo time = 3000/90 ms, field of view (FOV) = 128×128 mm^2^, matrix = 106×106, with in-plane interpolation into 212×212, slice thickness = 2.4 mm, slices = 12, averages = 16, phase partial Fourier = 7/8 and bandwidth = 1096 Hz/pixel. In order to maintain the consistency of slices among the 12 scans, the 9^th^ slice (craniocaudal direction) of each scan was positioned at the junction between the pons and the cerebral peduncle on mid-sagittal T2W image and perpendicular to the long axis of brain stem.

### Data preprocessing

Eddy current and movement corrections were performed using FSL 4.1 (FMRIB Software Library, The University of Oxford, UK). Then the diffusion tensor of each voxel was calculated based on a Gaussian diffusion signal decay model as well as linear least-squares fits. The three eigenvalues (λ1, λ2, and λ3) were extracted by diagonalization of the diffusion tensor [35]. Finally, the MD, FA, and λ23 were calculated by the three eigenvalues according to the following equation.
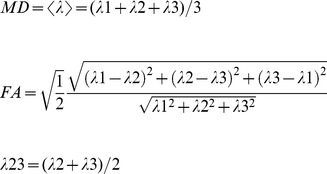
(1)


All maps of diffusion indices except for λ23 were acquired using FSL 4.1. The λ23 was calculated with Matlab 2009a (The MathWorks, Inc., Natick, Massachusetts, USA).

### ROI definition

**Figure 2:**
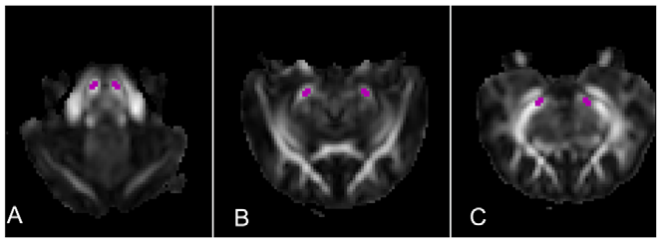
ROI definition of corticospinal tract. Three sections of corticospinal tract were defined on FA maps at 8^th^ day after modeling, including the pons (A), cerebral peduncle (B), and internal capsule (C) level, respectively. Note the decreased FA signal at the left CST.

**Figure 3:**
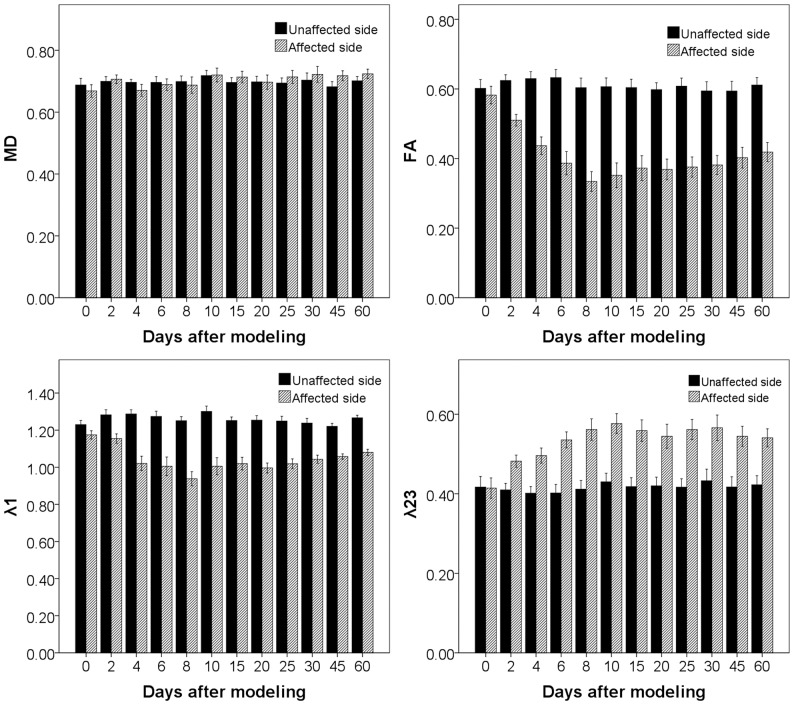
Dynamic changes of diffusion indices. The diffusion indices on the intact side of corticospinal tract (black bar) showed little changes between each time points, which however, evolved dynamically on the injury side (twill bar).

Regions of interest (ROIs) were defined using the software of the MRICRON (http://www.cabiatl.com/mricro/mricron/index.html) according to the following procedures: (1) a multi-contrast method [31] combined with different diffusion indices was chosen to define ROIs at the affected and unaffected CSTs; (2) three sections of CST were measured at the levels of the internal capsule, cerebral peduncle, and pons, respectively (**Fig. 2**); (3) to minimize partial volume effect, the size of ROIs was restricted to the range from 6 to 8 pixels, which were comparable with the size of CST on the transverse sections; (4) the selected sections were kept as same as possible among the 12 scans. In each scan, the location, size, and shape of the ROI at the affected side of the CST were consistent with those at unaffected side; (5) the defined ROIs were copied to each parametric map of diffusion indices. Finally the FA, MD, λ1 and λ23 values of the ROIs were extracted; (6) two raters (M. Z. and W. Q.) drew ROIs independently based on the above criteria to test the inter-rater reliability of ROI definition. Intra-class correlation coefficients (ICC) showed considerably high reproducibility between the two raters (ICC = 0.957,*P*<0.001). The mean values of the two measurements by the two raters were selected for further statistical analysis.

### Pathological examinations

Pathological examinations included Hematoxylin and Eosin (H&E) stain, Luxol fast blue (LFB) stain, and Immunohistostain with primary antibodies against neurofilament protein (NF; Zymed, San Francisco, CA, USA; 1∶100).

After intraperitoneally injected a lethal dose of sodium pentobarbital (100 mg/kg), the animals immediately underwent thoracotomy and laparotomy. Then the abdominal aorta was clamped with a hemostatic forceps, and 2000 ml physiological saline (P.S.) was immediately perfused into the ascending aorta, followed by 2000 ml 4% paraformaldehyde fixative in 0.1 M sodium phosphate buffer (pH 7.4). Subsequently, the skulls were removed and the whole brain tissues including medulla oblongata were extracted and immersed into the fixative for about 4 weeks.

Three 5 mm thick pieces were extracted from the fixed cat brain, which roughly corresponded to the MR slices containing the ROIs. The specimens were dehydrated with graded ethanol and then embedded in wax. Sections with a 5 µm thickness were obtained and stained with H&E, LFB and primary antibodies against NF.

### Statistical analysis

The data were analyzed with PASW statistics 18 (SPSS Inc, Chicago, Ill). To study the stability of diffusion indices during the observing time window, a one-way repeated measurement analysis of variance (ANOVA) was performed to test the differences in diffusion indices of the unaffected CST among the 12 scans. In order to minimize the effects of system drift on diffusion indices among the 12 scans, we calculated the ratios of diffusion indices (rFA, rMD, rλ1 and rλ23) between the ROIs of affected and unaffected sides, and performed a one-way repeated measurement ANOVA on the rFA, rMD, rλ1 and rλ23 of the CSTs among the 12 scans. Least significant difference (LSD) method was used to perform the *post hoc* analyses to examine the differences between every 2 TPs. During the late stage of WD, diffusion indices underwent slow fluctuations. Pearson correlation coefficients were selected to sensitively determine which diffusion index linearly changes with time from 8 to 60 days after injury. The significance level was defined as *P*<0.05 (2-tailed) for all statistical procedures.

## Results

### The reproducibility of diffusion indices across scans

At the unaffected CST, repeated measurement ANOVA showed no significant differences in the FA (F value = 1.27, *P* = 0.29), MD (F value = 0.64, *P* = 0.64), λ1 (F value = 1.97, *P* = 0.11) and λ23 (F value = 0.63, *P* = 0.64) between the 12 TPs (**Fig. 3**). This suggested high reproducibility of diffusion indices among the TPs.

### The dynamic evolution of diffusion indices of the degenerated CST

**Figure 4:**
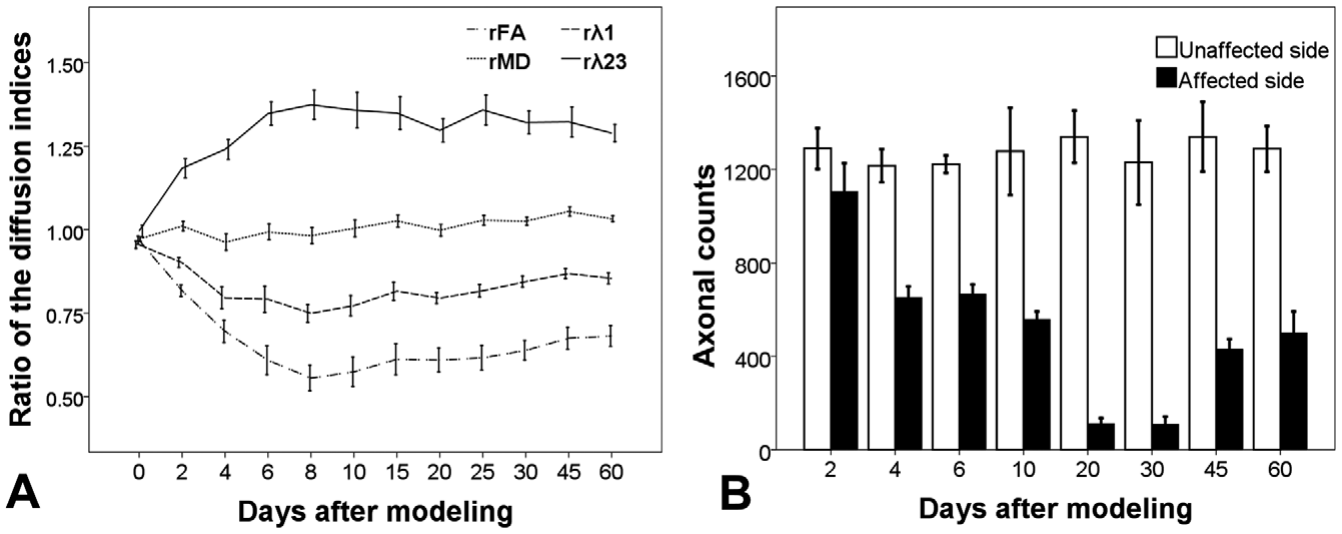
Correlation between the ratios of diffusion indices and the axonal counts. Panel A showed the dynamic changes of the ratios of diffusion indices, and Panel B showed the corresponding axonal counts at the injury side. Note a significant increase of axonal counts at 45^th^ and 60^th^ day after modeling.

The ratios of diffusion indices (rFA, rMD, rλ1 and rλ23) between the affected and unaffected sides of ROIs were used to show the dynamic changes during the process of CST degeneration. A one-way repeated measurement ANOVA showed significant changes across the 12 scans in the rFA (F = 36.7, *P*<0.001), rMD (F = 2.48, *P* = 0.008), rλ1 (F = 11.74, *P*<0.001), and rλ23 (F = 14.64, *P*<0.001). Further *post hoc* analyses (LSD method) revealed significant decreases in rFA (*P*<0.001) and rλ1 (*P* = 0.02), and increase in rλ23 (*P*<0.001) at the 2^nd^ day compared with those at baseline (**Fig. 4A**).

Following the initial changes at the 2^nd^ day, rFA and rλ1 quickly dropped into the minimal values (0.556±0.134 and 0.750±0.091, respectively) and rλ23 rose to the peak (1.348±0.120) at the 8^th^ day after injury (**Fig. 4A**). Then slight increases of rFA (r = 0.298, *P* = 0.003), rλ1 (r = 0.405, *P*<0.001) and rMD (r = 0.250, *P* = 0.014) were shown from 8^th^ day to 60^th^ day, while rλ23 did not show any statistical changes (r = −0.148, *P* = 0.15). According to the patterns of evolution of diffusion indices, Wallerian degeneration of CST can be divided into two stages: (1) Early stage - the first 8 days after injury, manifested as sharply decreased FA and λ1, increased λ23 and unchanged MD. (2) Late stage - 8 days later after injury, manifested as slightly increased FA, MD and λ1, and unchanged λ23 (**Fig. 4A**).

### The pathological processes of the degenerated CST

#### Axonal changes

**Figure 5:**
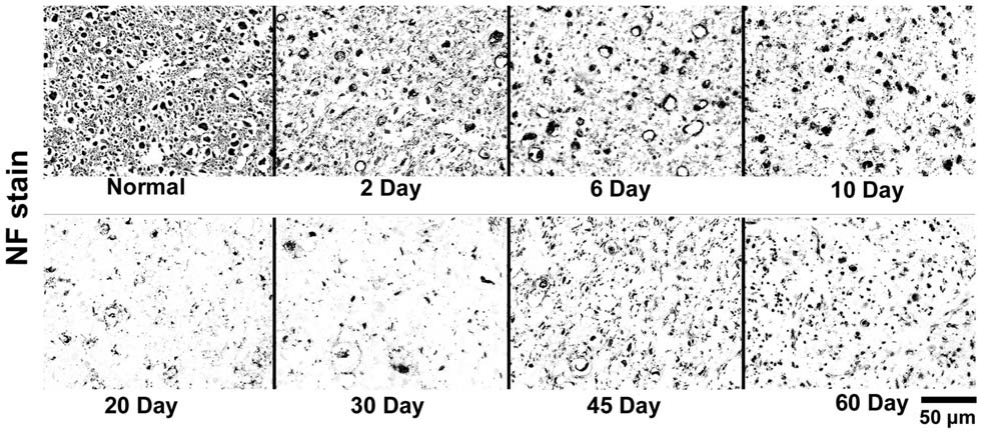
NF stained histological images of cat's pons. The NF images of the normal side and the degenerated side of pons at 2^nd^, 6^th^, 10^th^, 20^th^, 30^th^, 45^th^ and 60^th^ day after modeling were displayed. Scale bar, 50 µm.

Axonal degeneration was detected at the 2^nd^ day after cortical resection, which was characterized by a few massive swollen axons on NF and H&E stains. Some of them disintegrated and aggregated around the axonal membranes, forming vacuole-like structures. At the 6^th^ day, the amount of swollen and vacuolar axons was increased, and some of them were degraded into dark-stained fragments. At the 10^th^ day, the degenerated axons manifested as atypical dark-stained debris on NF stain with little swollen or vacuolar changes. At the 20^th^ day and the 30^th^ day, there were few positively stained structures in the whole visual field. Interestingly, a few small axons emerged at the 45^th^ day and the 60^th^ day (**Fig. 4B** and **Fig. 5**).

#### Microglia activation

**Figure 6:**
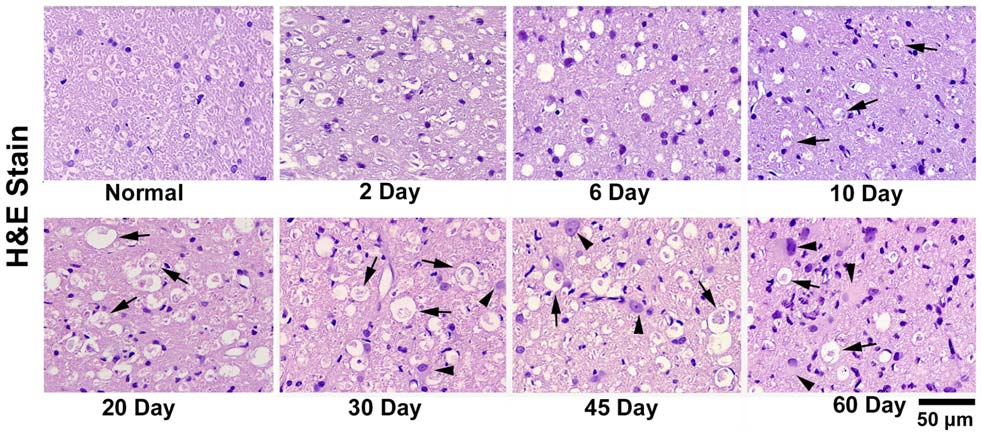
H&E stained histological images of cat's pons. The H&E images of normal side and the degenerated side of pons at 2^nd^, 6^th^, 10^th^, 20^th^, 30^th^, 45^th^ and 60^th^ day after modeling were displayed. Black arrows indicated the activated microglias, and the black arrow heads labeled the reactive astrocytes. Microgliosis emerged at 10^th^ day after modeling, while marked astrocytosis started at 45^th^ day after modeling. Scale bar, 50 µm.

**Figure 7:**
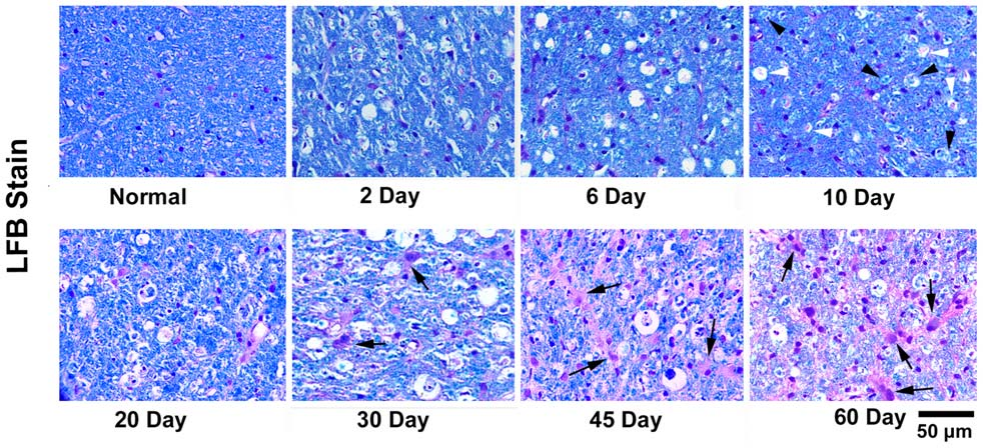
LFB stained histological images of cat's pons. The LFB images of normal and degenerated side of pons at 2^nd^, 6^th^, 10^th^, 20^th^, 30^th^, 45^th^ and 60^th^ day after modeling were displayed. White arrow heads indicated the activated microglias that phagocytized the axonal debris (purple stained), while black arrow heads labeled the microglias with myelin debris (blue stained). Black arrows indicated the reactive astrocytes. Scale bar, 50 µm.

The initial proof of microglia activation was found at the 10^th^ day after modeling on H&E and LFB stains. At this time point, H&E staining showed a few irregular ramified cells, which contained atypical ingredients being stained as purple by H&E and these atypical ingredients were stained as blue (indicating myelin debris) or purple (indicating axon debris) On LFB, implying activated microglia. Microglia activation became more predominant at the 20^th^ day, which had the typical round, foamy appearances and marked phagocytizing activities. Microglia kept being activated during the total observation time window (**Fig. 6** and **Fig. 7**).

#### Myelin clearance

**Figure 8:**
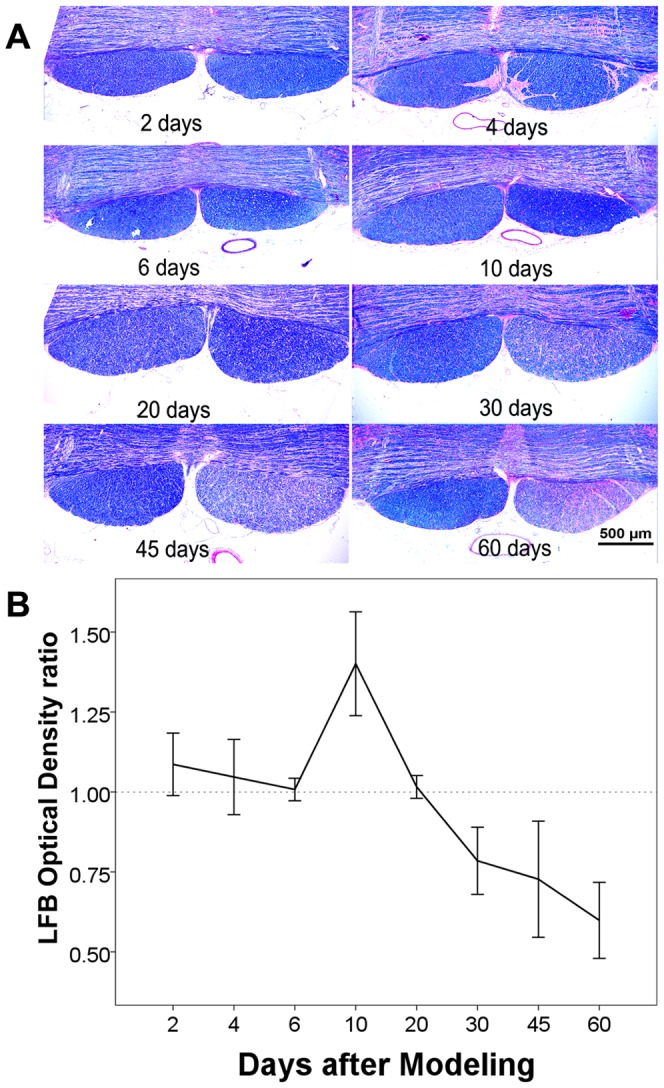
Quantitative analysis of LFB stain. (A) LFB stained histological images of the pons at 2^nd^, 4^th^, 6^th^, 10^th^, 20^th^, 30^th^, 45^th^ and 60^th^ day after modeling. Scale bar, 500 µm. (B) Time course of the LFB optical density ratios between the degenerated and normal sides of CST. Progressive decreases of optical density were shown 20 days after modeling, implying the clearance of myelin debris.

LFB is a traditional histological stain for myelin sheath, which stains myelin ingredients with blue and nerve cells with purple. From the 2^nd^ day to the 6^th^ day, LFB stain did not reveal any differences in coloration between the affected and unaffected CSTs except for some vacuolar lucent areas. At the 10th day, the degenerated CST manifested darker blue coloration relative to the unaffected side on LFB. From the 20^th^ day to the 60^th^ day, blue coloration at the degenerated CST progressively became lighter, which suggested myelin clearance (**Fig. 7** and **Fig. 8**).

#### Astrocytosis

Another important pathological change during WD in CNS is astrocytosis. Astrocytes play principal roles in the repair and scarring process of the CNS following traumatic injuries. The reactive astrocytes were characterized by big light-stained nucleus and abundant eosinophilic (pink) cytoplasm on H&E stain. In present study, a large number of reactive astrocytes were not shown until the 45^th^ day after modeling (**Fig. 6**). The typical “star-shaped” purple-stained cells were also presented on LFB at this stage, which suggested the formation of astrocytic scar (**Fig. 7**).

## Discussion

**Figure 9:**
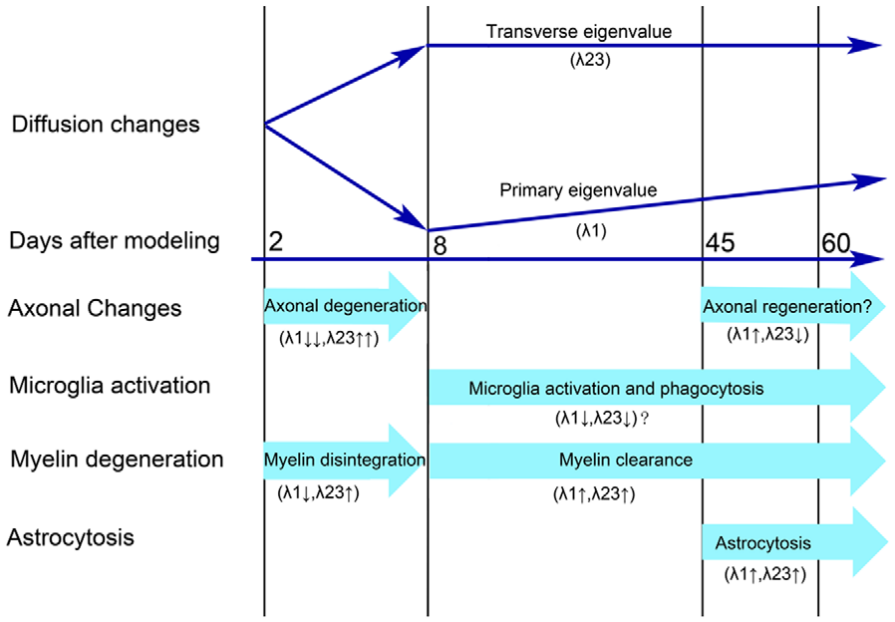
Schematic associations between the evolution of diffusion indices and underlying pathology in Wallerian degeneration.

In this study, we established a cat WD model to investigate the pathological bases underlying the dynamic evolution of diffusion indices in degenerated CST. We found quick decreases of FA, λ1 and increase of λ23 from the 2^nd^ day to the 8^th^ day after modeling, which was accompanied with progressive axonal disintegration. Afterwards, FA, MD and λ1 were slightly increased from the 8^th^ day to the 60^th^ day, while λ23 kept unchanged. The pathological changes of this stage were characterized by a series of processes, including microglia activation, myelin clearance, astrocytosis and possible axonal regeneration (**Fig. 9**). Our findings help to understand the pathological bases that result in the changes of diffusion indices of the degenerated CST, and suggest that DTI has the potential to monitor the pathological processes of WD in CNS *in vivo*.

The initial detectable diffusion changes in affected CST occurred as early as 2 days after cortical injury and the time scale was consistent with the findings revealed by pathological examinations. Our finding indicates that DTI is a sensitive method to monitor WD in CNS. In the early studies, significant decrease in FA and λ1 and increase in λ23 at the degenerated tract started as early as 3 to 14 days after insult [6], [18], [29], [31], [36], which also implied that the time scale of detectable diffusion changes in degenerated tract might be varied with species, injury types and degree, and so on. Thereafter, diffusion indices were quickly changed at the initial several days, and were slightly evolved for the remaining time points (**Fig. 4A**). This process was well consistent with initial findings that WD happened fast but evolves much slower in CNS (months to years) [4], [6], [37] than in PNS [3], [37], [38].

### The pathologic bases underlying the diffusion evolution of WD in CNS

In the following discussion, we just focused on the evolution of λ1 and λ23 since they are independent indices and the bases for calculating other diffusion indices, such as FA and MD [39]. Although the pathological processes of WD evolved continuously, we divided the time course of WD into two stages according to the diffusion changes in order to explore which pathologic processes were involved in each stage and how they contributed to the changes in diffusion indices (**Fig. 9**).

#### Early stage

At the early stage, the λ1 started to decrease at the 2^nd^ day after injury, and quickly dropped into the minimum value within a few days (**Fig. 4A**), which was accompanied with progressive axonal disintegration (**Fig. 4B** and **Fig. 5**). In a comprehensive review of the basis of anisotropic water diffusion in the nervous system, Beaulieu suggests that anisotropic water diffusion in neural fibers is mainly due to the dense packing of axons and their inherent axonal membranes, whereas myelin may make minor contribution by modulating the degree of anisotropy in a given fiber tract [40]. Based on this theory, axonal disintegration in this study destructed the orientation of the tract; furthermore, scrambled axonal and myelin debris would restrict the water diffusion along the tract, resulting in the decrease of λ1. This inference also supported by the hypotheses of previous authors [29], [30], who ascribed the decreased λ1 to axonal degeneration.

However, an important issue should be stated that the λ23 began to increase at the 2^nd^ day after injury and quickly reached the peak, which was highly corresponded with the time course of λ1. This finding was consistent with the report by Zhang et al [30], but much earlier than that (5 to 9 days) reported by Song et al [29], [31]. According to Song's reports, delayed increase of λ23 was closely correlated with the clearance of myelin basic protein, which constituted the basis of the hypothesis that λ23 is a good marker of myelin degeneration in WD. However, this hypothesis is not supported by the following pieces of evidence. First, the integrity of axonal membranes constitutes the major basis of diffusion anisotropy in nervous system [23], [40]. The disintegration of axons in the initial stage of WD theoretically can also result in the increase of the transverse diffusivity (λ23) because axonal membranes are broken down, which increases the water exchange between intra-axonal and extra-axonal space. Second, myelin disintegration happened soon after axonal degradation. Buss et al found that myelin associated glycoprotein (MAG), a peri-axonal myelin protein, has significantly decreased 3 days after injury [2]. The disintegration of myelin sheath (into myelin ovoid) further increase the water exchange between intra-axonal and extra-axonal space, as a result, water diffused more freely in directions perpendicular to the tract, resulting in the increase of λ23 [30]. Third, as shown in Figure 4 of Sun's report [31], the λ23 values of optical nerve and optical tract among 9, 14 and 28 days seemed unchanged (although not provide statistic results), just like the late stage in the present study. However, as shown in the present study (**Fig. 8**) and many other pathological findings [2], [3], [4], [37], myelin clearance occurred progressively during the WD course and was more prominent at the late stage. If Sun's hypothesis of λ23 representing the clearance of myelin debris is correct, the λ23 would have progressively increased, but this is not supported by their and our findings of unchanged λ23 at the late stage.

As a result, we hypothesized that λ23 also mainly reflected the axonal disintegration with minor contribution from the disintegration of myelin sheath at the early stage of WD in CST. At this stage, the axonal membrane is destroyed by calcium-dependent mechanisms [37], [41], [42]. The myelin sheaths also disintegrate into myelin ovoid soon after the breakdown of axonal membrane [30]. The disintegrations of axon and myelin sheath lead to the free exchange between intracellular and extracellular space, which means that the anisotropic basis in nervous system is destroyed. Thus, the λ23 was dramatically increased (by 37% in this study) at the initial 8 days after injury.

#### Late stage

From 8 to 60 days, the λ1 was slightly increased while the λ23 was unchanged (**Fig. 4A**). This process was accompanied with progressive microglia activation, myelin clearance and astrocytosis (**Fig. 6** **–** **8**). The differential changes of diffusion indices at this stage compared with the early stage indicated the termination of axonal disintegration, which also implied that the remaining pathological changes such as myelin clearance and astrocytosis might play a minor role in the diffusion changes at the degenerated CST. The clearance of myelin debris might diminish the hindrance to water diffusion, resulting in the slight increase of both λ1 and λ23. The preliminary evidence suggested that the formation of astrocytic scar had similar effects on water diffusion [43]. However, the relative unchanged λ23 in the present study implied that there should be other pathologic processes contributing to the observed changes in water diffusion.

It was intriguing to note that a slight recovery of axonal counts was observed at the 45^th^ day after injury and later (**Fig. 4B** and **Fig. 5**). Although several myelin-associated inhibitors, such as myelin-associated glycoprotein [44] and NogoA [45], can result in the difficulty in axonal regeneration during the WD in CNS, spontaneous axonal regenerations in CNS were reported *in vivo* [46], [47], [48], [49], [50]. In CNS, axonal regeneration can be achieved either by immigration of new neuronal cells from precursor populations (neurogenesis) or by sprouting of surrounding un-injured axons (axonogenesis) [51]. In this study, the increased axonal sections after 45^th^ day than those at the 20^th^ or 30^th^ day after injury might be caused by following factors: (1) the axonal regrowth of injured axons; (2) rewiring of surviving or neighboring axonal connections. Lehmann et al reported that the observed diffusion indices were correlated with the total number of regenerating axons in PNS [52]. Similarly, if the axonal regrowth really exists in this study, the directionality of CST can be partially recovered, resulting in the increase of λ1 and decrease of λ23. However, because of limited experiment conditions in this study, we can not provide direct histopathological or immunostaining evidence of axonal regeneration in CNS, and the relationship between diffusion indices changes and axonal regeneration in CNS should be clarified in the future.

### Methodological considerations

In this study, we chose the CST of mature domestic cat as the research focus, which is mainly based on the following considerations: (1) the most popular and reliable DTI protocol is the SS-EP method. The major shortcoming of this method is susceptibility-induced image distortion, which is especially severe in small animals such as the rat's brain. In contrast, domestic cat has relative large brain volume (about 30 gram). The preliminary experiment showed relatively high quality in acquired diffusion images. (2) The CST is well developed in cat brain, and its origin cortex had been identified by senior researchers [32], which helps to establish a successful WD model. After modeling, all of the sections at the injured side showed significant axonal degeneration after 2 days and later while the contralateral CST remained intact, which suggested a successful WD model (**Fig. 5**). But several limitations about WD modeling should be mentioned: (1) specific markers such as anti-GFAP, anti-MBP and CD68 are very important for quantification the astrogliosis, myelin or microglia infiltration. However, we cannot obtain suitable markers that can specifically bind with nervous tissue of cats because of limited experiment conditions. As a result, we had to choose traditional stain methods (such as NF, H&E and LFB stain) to roughly display and evaluate the pathologic process of WD after injury. (2) Several experimental manipulations may improve our understanding on the relationship between diffusion indices representations of the pathologic process of WD in CNS. For example, what happens to DTI values when the axons are prevented from degenerating after injury (such as slow Wallerian degeneration mice)? And what happens to the DTI values of animals that are deficient in myelin (such as shiverer mice, which exhibit progressive demyelination)? However, the available experimental techniques and MRI equipments prevent us from performing these experiments at current stage. Future studies should be done to answer these questions.

In order to further improve the quality of diffusion images, a series of parameter optimization were performed on the DTI protocols: (1) thirty non-collinear diffusion gradients were used to ensure better fit of the diffusion tensor; (2) iPAT technique (acceleration factor = 4, reference lines = 24) further diminished the susceptibility induced image distortion; (3) the FOV was set as small as the size of cat's brain to increase the effective collecting volume; (4) a series parameters (including the bandwidth, matrix, averaging, slice thickness, etc) were optimized to ensure good signal-to-noise ratio and acceptable spatial resolutions. The average SNR of the ROI on the acquired b0 image was about 58.6±8.2, which was much higher than the reported value that can neglect the contamination of noise on diffusion indices [53].

Another issue is the extraction of the diffusion indices of the CST. Because cat's brain was much smaller compared with the human experiment, the whole CST was not possible extracted using fiber tracking method. A recent report by Yu et al showed that the ROI method was in good consistency with the fiber tracking method in quantification of diffusion indices of the WD at CST [6]. In this study, three ROIs at the level of internal capsule, cerebral peduncle, and pons were defined (Fig. 2). Then, two raters independently drew ROIs to decrease the subjective error. ICC analysis showed high reproducibility (ICC = 0.957, *P*<0.001) between the two raters. Furthermore, as shown in Figure 8, the section of the injured CST did not show significant atrophy during the WD process, indicating that ROI definition cannot be influenced by possible atrophy caused by WD. At last, in order to minimize the influence of system drift of diffusion indices among the 12 scans, we used the ratios of diffusion indices (rFA, rMD, rλ1 and rλ23) between the affected and unaffected ROIs to study the dynamic changes of the diffusion indices in WD. A prerequisite of this ROI method is that there are no plastic changes at the healthy side of the CST during the follow-up period, which had been confirmed by Yu et al [6] and by the present study.

This study is ineluctably compromised by the partial volume effect. Compared with the size of CST section, the voxel size (about 0.36 mm2 in slice) of each ROI was relative large, which might include unwanted non-CST structure in the ROI definition. Several efforts (such as small ROI, multi-contrast ROI selection, and inter-rater test etc.) have been done to diminish the effect. But the most optimal method, smaller voxels, was compromised by another more important factor, the image SNR. Further studies are needed to verify our findings.

## Conclusions

In the present study, we showed that DTI can detect WD in CST as early as 2 days after cortical injury, which was consistent with the pathological findings. We also systematically investigated the relationships between the dynamic changes of diffusion indices and the underlying pathological processes during the evolution of WD in CNS, which may contribute to the understanding the changes of diffusion indices in the context of pathological aspects. It should be emphasized that our findings are specific to an acute WD model and should not be generalized to all situations in humans, especially the chronic neurodegenerative diseases, such as Alzheimer's disease.
